# Pediatric brain arteriovenous malformation unfavorable hemorrhage risk: extrapolation to a morphologic model

**DOI:** 10.1186/s41016-018-0123-x

**Published:** 2018-07-02

**Authors:** Zongze Li, Li Ma, Chunxue Wu, Jun Ma, Xiaolin Chen

**Affiliations:** 1 Department of Neurosurgery, Peking University International Hospital, Beijing, 102206 People’s Republic of China; 2 Department of Neurosurgery, Beijing Tiantan Hospital, Capital Medical University, No. 6 Tiantan Xili, Dongcheng District, Beijing, People’s Republic of China; 3 China National Clinical Research Center for Neurological Diseases, Beijing, People’s Republic of China; 4 Department of Neuroradiology, Beijing Tiantan Hospital, Capital Medical University, Beijing, People’s Republic of China; 5 Center of Stroke, Beijing Institute for Brain Disorders, Beijing, People’s Republic of China; 6 Beijing Key Laboratory of Translational Medicine for Cerebrovascular Disease, Beijing, People’s Republic of China

**Keywords:** Arteriovenous malformation, Children, Hemorrhage, Risk prediction

## Abstract

**Background:**

Children with brain arteriovenous malformations (bAVM) are at risk of life-threatening hemorrhage contributing to unfavorable neurological deficit in their early lives. Our aim was to propose a classification system predicting the unfavorable hemorrhage in children with bAVM.

**Methods:**

We identified all consecutive children admitted to our institution for bAVMs between July 2009 and August 2015. A hemorrhage event was defined as unfavorable when it is life-threatening (requiring emergent invasive intervention) or with post-hemorrhage mRS > 3. The effects of demographic characteristics and bAVM morphology on unfavorable hemorrhage risk were studied using univariate and multivariable regression analyses, followed by discrimination analysis using area under the receiver operating curve (AUROC) and 5-fold cross validation.

**Results:**

A total of 162 pediatric bAVM cases were identified, unfavorable hemorrhage occurred in 49 (30.2%). Periventricular nidus location (HR, 4.46; 95%CI, 1.93–10.31; *P* < 0.001), non-temporal lobe location (HR, 2.72; 95%CI, 1.20–6.15; *P* = 0.02) and long pial draining vein (HR, 3.26; 95%CI, 1.53–6.97; *P* = 0.002) were independent predictors of an earlier unfavorable hemorrhage in pediatric bAVMs. We further classified the bAVM into three types: Type I, periventricular and non-temporal location (Ia, deep location; Ib, superificial location); Type II, with long pial draining vein and non-periventricular or temporal location; Type III, non-periventricular or temporal location without long draining vein. Predictive accuracy of this classification for unfavorable hemorrhage was assessed with AUROC of 0.77 (95% CI 0.69–0.85) and remained stable after cross validation.

**Conclusion:**

A morphologic model based on nidus location and venous drainage might predict unfavorable hemorrhage in children with bAVM.

## Background

Children with brain arteriovenous malformations (bAVM) are more commonly with a hemorrhagic presentation [1–3]. bAVM rupture is associated with a 5–10% chance of death and a 30–50% chance of permanent or disabling neurological deficit [4]. Therefore, what the children’s family and health care professionals really concern is the risk of life-threatening and disabling hemorrhage. A recent study in pediatric bAVM patients indicated long-term poor outcome after treatment was significantly correlated with modified Rankin Scale (mRS) score after hemorrhage, rather than hemorrhagic presentation [5]. Previous reports revealed that 20–26% of children with bAVM experienced disabling neurological deficits after hemorrhage [5–8]. Given the prognosis correlating with post-hemorrhage neurological function and catastrophic impact of disabling neurological deficit in children, it is important to identify risk factors that can be used to predict unfavorable hemorrhage.

This study aimed to: (1) assess the association of morphologic features with the risk of unfavorable hemorrhage in children with bAVMs; (2) establish risk stratification models predictive of unfavorable in children with bAVM.

## Methods

The study protocol was approved by the Institutional Review Board of our institution. Written informed consent was obtained from all participants and their guardians at admission.

### Patients and study design

The AVM database at our institution has been previously described and is a prospectively maintained database collecting demographic, clinical, and neuroradiological data for all patients with a confirmed angiographic or histological diagnosis of intracranial AVM treated at our institute. [7, 9] This database was reviewed to identify all consecutive children with bAVMs (≤18 year-old at the first angiographic diagnosis of bAVM) between July 2009 and August 2015.

Neurological function was assessed using mRS. Post-hemorrhage outcome was defined as unfavorable: (1) mRS score > 3 or (2) undergoing emergent invasive intervention. The immediate post-hemorrhage mRS were recorded within 24 h after hemorrhage. A clinician who was not directly involved in the care of these patients performed all scale assessments. Emergency intervention for ICH evacuation or drainage was considered in patients with GCS ≤ 8, ICH with midline shift or hydrocephalus.

### Neuroradiological review

MR, CT and angiographic images available for each patient were evaluated by consensus between two experienced neuroradiologists (J.M. and C.W.) who were blinded to the clinical information. A structured list of angiographic and MR features (location, size, venous drainage and arterial supply) was retrospectively scored using a protocol that generally conformed to the consensus recommended by a Joint Writing Group for bAVM research reporting terminology. [10] bAVM location was further dichotomized into deep (basal ganglia, thalamus, cerebellum, and corpus callosum) and superficial (all other locations). A posterior fossa location was defined as brainstem, cerebellum, or both. Eloquent brain was defined as previously reported in Spetzler-Martin grading system. bAVMs were also classified as having a periventricular location if the nidus (with a contrast-enhancement or flow void) contacted the ependymal lining of the ventricle on contrast-enhanced T1- and T2-weighted images as we previously described. [7, 11–13] Venous drainage was dichotomized into exclusively deep venous drainage or non-exclusively deep venous drainage (superficial-only drainage or superficial and deep drainage). Long pial draining vein was defined as a superficial draining vein with a length longer than 3 cm [14]. Associated aneurysms only included aneurysms related to shunt flow. For statistical analysis, the associated aneurysm variable was dichotomized into absent or present.

### Statistical analysis

Data were analyzed using IBM SPSS Statistics Version 22.0 (IBM, Armonk, New York) and MedCalc Version 18.5 (MedCalc software, Ostend). Statistical significance was set at *P* < .05. For the neuroradiological and clinical data, patients with and without unfavorable hemorrhage were compared using descriptive statistics, including t-tests for continuous variables and *χ*^*2*^ tests for categorical variables.

We examined the association of potential predictors with the time from birth to unfavorable hemorrhagic event, censoring patients at the time of treatment of bAVM or last follow-up. Kaplan-Meier survival curves, log-rank tests were used. Multivariable Cox regression model was established including the potential predictors that have been reported to be predictive of hemorrhage in previous studies, as listed in Table 1. All the variables were entered and adjusted relative risks were calculated for all of them accordingly by the stepwise procedure (adjusted model with main effects).

**Table 1:** Association of Potential Predictors with Unfavorable Hemorrhage-free Survival before Treatment^a^

Characteristic	Univariable Cox Proportional Hazards (*n* = 162)			Multivariable Cox Proportional Hazards^b^ (*n* = 162)		
	HR	95%CI	*P* value	HR	95%CI	*P* value
Periventricular location	2.88	1.41–5.89	0.004	4.46	1.93–10.31	< 0.001
Non-temporal lobe location	2.26	1.04–4.90	0.039	2.72	1.20–6.15	0.016
Long pial draining vein	1.75	0.93–3.28	0.083	3.26	1.53–6.97	0.002
Maximal AVM size (mm)	0.99	0.98–1.01	0.484	0.99	0.97–1.01	0.213
Gender, male	0.83	0.44–1.54	0.546	0.90	0.44–1.84	0.778
Deep location	1.32	0.71–2.45	0.377	0.78	0.35–1.76	0.553
Posterior fossa location	1.28	0.39–4.17	0.682	2.01	0.51–7.96	0.323
Eloquence	0.79	0.43–1.47	0.456	0.68	0.34–1.38	0.286
Exclusively deep venous drainage	1.72	0.72–4.09	0.220	2.80	1.01–7.79	0.048
Associated aneurysm	2.05	0.80–5.28	0.137	1.63	0.55–4.86	0.377

^a^*P* value in boldface indicates statistical significance

^b^Adjusted for gender, deep location, infratentorial location, eloquence, associated aneurysm, maximal nidus size

**Fig. 1:**
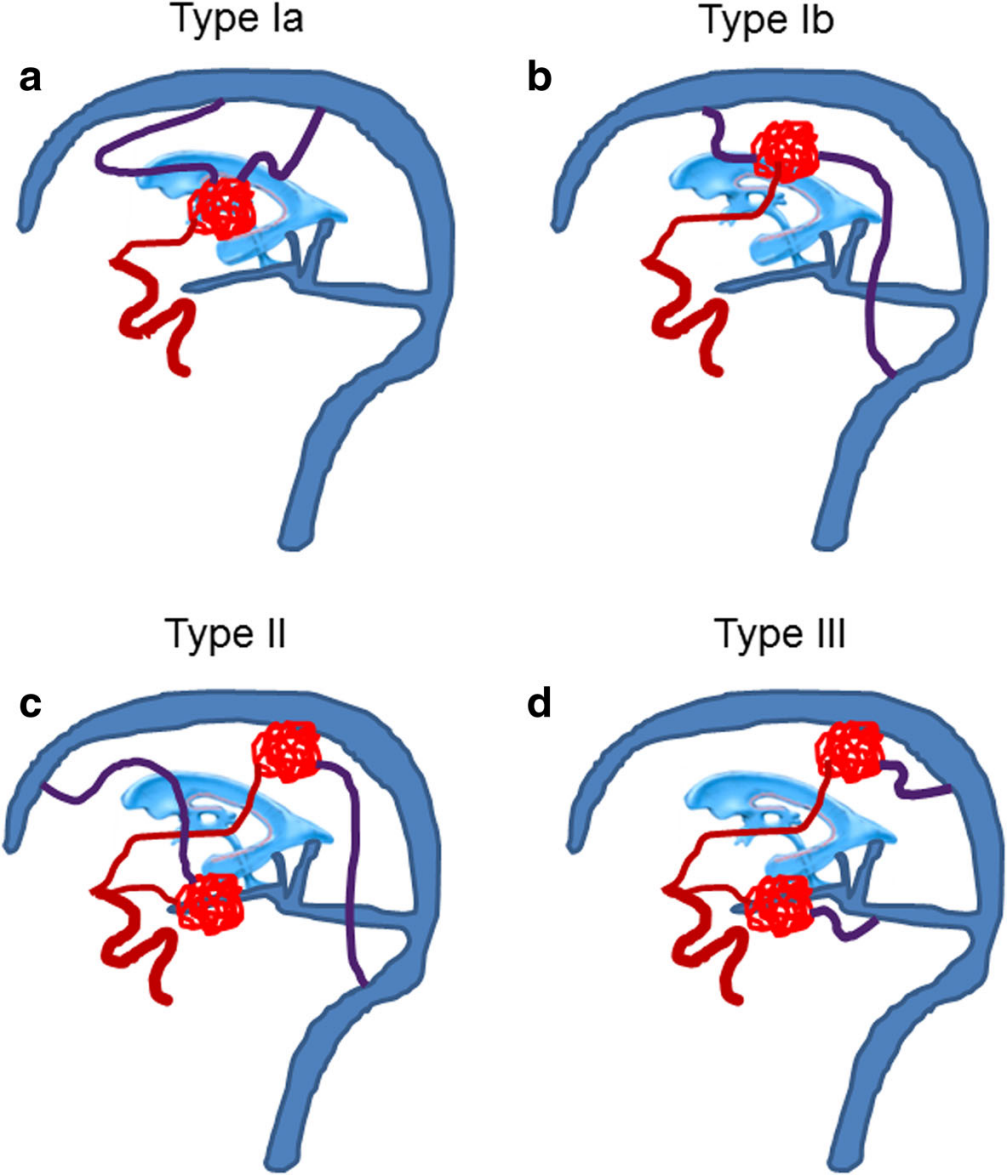
bAVM morphologic model predictive of unfavorable hemorrhage. Type I based on periventricular and non-temporal location with or without long draining vein (purple color) (**a** and **b**). Subtype Ia based on corpus callosum, basal ganglia or thalamus adjacent to ventricle; Subtype Ib were based on frontal, parietal, occipital lobe or cerebellum adjacent to ventricle; Type II (**c**) were with long pial draining vein and based on non-periventricular location (frontal, parietal or occipital lobe or deep location) or temporal lobe; Type III (**d**) were also based on non-periventricular location or temporal lobe but without long draining vein

With the multivariable analysis, we proposed three stratification models to predict unfavorable presentation: (1) full regression variable model; (2) concise model including significant risk factors from multivariable analysis and (3) AVM classification based on location and venous drainage type: Type I, periventricular and non-temporal location (Ia, deep location; Ib, superificial location); Type II, with long pial draining vein and non-periventricular or temporal location; Type III, non-periventricular or temporal location without long draining vein (Fig. 1). Receiver operating characteristic (ROC) analyses were performed, and the area under the receiver operating curve (AUROC) was compared using pairwise comparison of ROC curves, for discrimination of different models in predicting post-hemorrhage outcome. Five-fold cross validation was further used to evaluate whether the predictive accuracy of different models was overly optimistic. The data were divided into 5 equal subgroups; the model was then fitted 5 times to different combinations of 4 of the 5 subgroups and used to predict post-hemorrhage outcome in the remaining one-fifth of the data.

## Results

A total of 162 pediatric patients with bAVM were identified in 997 patients with a confirmed angiographic or histological diagnosis of intracranial AVM. None of the patients had familial bAVM or hereditary hemorrhagic telangiectasia. Baseline characteristics are shown in Table 2. Unfavorable hemorrhage occurred in 49 of 162 patients (30.2%). The majority of the patients experienced an unfavorable hemorrhage during the first 13 years of life (39/49; 79.6%).

**Table 2:** Baseline Characteristics of BAVM by Unfavorable or No unfavorable hemorrhage^a^

Characteristic	No unfavorable hemorrhage (*n* = 113)	Unfavorable hemorrhage (*n* = 49)	Total (*n* = 162)	*P* value
Demographic				
Gender				.36
Female	42 (37.2)	22 (44.9)	64 (39.5)	
Male	71 (62.8)	27 (55.1)	98 (60.5)	
Age at diagnosis (ys)	12.66 ± 4.00	11.59 ± 3.43	12.33 ± 3.85	.14
Clinical				
mRS at presentation				< .001^c^
0	11 (9.7)	0 (0)	11 (6.8)	
1	53 (46.9)	0 (0)	53 (32.7)	
2	27 (23.9)	1 (2.1)	28 (17.3)	
3	22 (19.5)	3 (6.1)	25 (15.4)	
4	0 (0)	20 (40.8)	20 (12.4)	
5	0 (0)	25 (51.0)	25 (15.4)	
Radiological				
Supratentorial location	107 (94.7)	45 (91.8)	152 (93.8)	.74^b^
Deep location	36 (31.9)	23 (46.9)	59 (36.4)	.08
Eloquence	55 (48.8)	22 (44.9)	77 (47.5)	.66
Periventricular location	55 (48.7)	37 (75.5)	92 (56.8)	.002
Temporal lobe location	44 (38.9)	10 (20.4)	54 (33.3)	.02
Maximal nidus size (mm)	11.49 ± 4.21	39.13 ± 19.96	39.49 ± 18.73	.88
Exclusively Deep Venous drainage	10 (8.8)	7 (14.3)	17 (10.5)	.30
Long pial draining vein	49 (43.4)	30 (61.0)	79 (48.8)	.04
Associated aneurysm	11 (9.7)	6 (12.2)	17 (10.5)	.63

^a^Table entries are No. (%) or mean ± SD. *P* value in boldface indicates statistical significance

^b^*P* values are from the *χ*^2^ test (correction for continuity)

^c^*P* values are from the Mann-Whitney U test

### Association of Morphologic Features with unfavorable hemorrhage-free survival

Assuming that the BAVM had been present since birth, there were 2025 patient-years of follow-up for this population of 162 patients (mean ± SD, 12.5 ± 4.6 years). A total of 49 unfavorable hemorrhage occurred in children with BAVM, yielding an overall annual rate of unfavorable hemorrhage of 2.4% for pediatric patients.

**Fig. 2:**
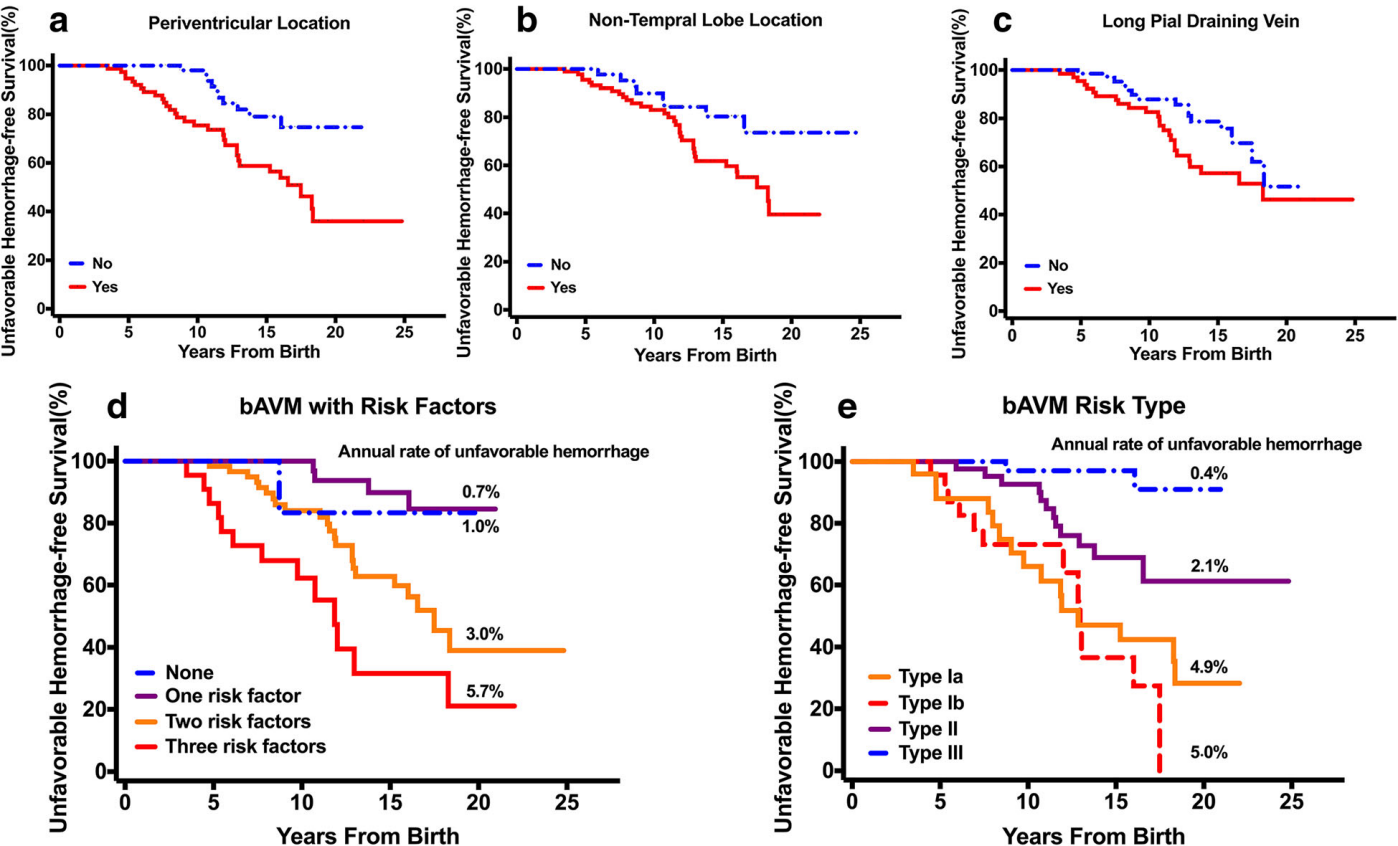
Kaplan-Meier curves demonstrating unfavorable hemorrhage-free survival difference between bAVMs with and without certain morphologic features. **a** There was a significant difference between the time to unfavorable hemorrhage for patients with periventricular and non-periventricular bAVMs (log-rank, *P* = .002). **b** There was also a significant difference between the time to unfavorable hemorrhage for patients with temporal location and non-temporal bAVMs (log-rank, *P* = .03). **c** Children with non-temporal bAVMs tended to present unfavorable hemorrhage earlier than non-temporal lobe bAVMs (log-rank, *P* = .08). **d** The unfavorable-hemorrhage free survival was similar in children with or without one biomarker. In contrast, children harboring two or more biomarkers were at higher risk for early unfavorable hemorrhage (log-rank, *P* < .001). bAVMs were further classified into four types with these features. **e** Children with Type Ia and Ib lesions presented with unfavorable hemorrhage earlier than those with Type II lesions (log-rank, *P* < .001)

There was a significant difference between the time to unfavorable hemorrhage for patients with periventricular and non-periventricular BAVMs (log-rank, *P* = 0.002). Children with periventricular BAVMs present unfavorable hemorrhage earlier, with a median unfavorable hemorrhage-free survival of 17.49 years (95 %CI, 14.45–20.53) for periventricular BAVMs (Fig. 2). There was also a significant difference between the time to poor outcome before treatment for patients with temporal location and non-temporal BAVMs (log-rank, *P* = 0.034). Children with non-temporal BAVMs present poor outcomes earlier, with a median poor outcome-free survival of 18.28 years (95 %CI, 15.86–20.70) for non-temporal lobe BAVMs. The association is not significant between the presence of long pial draining vein (log-rank, *P* = 0.079) or exclusively deep venous drainage (log-rank, *P* = 0.214).

Both univariate and multivariable Cox regression analyses were performed on the 162 pediatric patients (Table 3). Both the univariate analysis identified a periventricular nidus location (HR, 4.46; 95%CI, 1.932–10.31; *P* < 0.001) and non-temporal lobe location (HR, 2.72; 95%CI, 1.20–6.15; *P* = 0.02) as predictors for future poor outcome in pediatric BAVMs and long pial draining vein (HR, 3.26; 95%CI, 1.53–6.97; *P* = 0.002) were significantly associated with earlier unfavorable hemorrhage in the multivariable model adjusting for other characteristics.

**Table 3:** BAVM morphologic classification associated with unfavorable hemorrhage risk^a^

Characteristics	Type Ia (*n* = 30)	Type Ib (*n* = 29)	Type II (*n* = 52)	Type III (*n* = 51)
Morphologic Features				
Nidus location	Periventricular and non-temporal (corpus callosum, basal ganglia or thalamus)	Periventricular and non-temporal (frontal, parietal, occipital lobe or cerebellum)	Non-periventricular or temporal lobe	Non-periventricular or temporal lobe
Long pial draining vein	13 (43.3)	14 (48.3)	52 (100.0)	0 (0)
Hemorrhage				
mRS at bAVM rupture				
≤ 2	9 (30)	12 (41.4)	30 (57.7)	41 (80.4)
>2	21 (70)	17 (58.6)	22 (42.3)	10 (19.6)
Unfavorable hemorrhage				
No	12 (40.0)	15 (51.7)	38 (73.1)	48 (94.1)
Yes	18 (60.0)	14 (48.3)	14 (26.9)	3 (5.9)
Follow-up period, patient-years	367	280	667	711
Annual rate of unfavorable hemorrhage, %	4.9	5.0	2.1	0.4
Spetzler-Martin Grade				
I-II	7 (23.3)	20 (70.0)	30 (57.7)	29 (56.9)
III	11 (36.7)	6 (20.7)	14 (26.9)	18 (35.3)
IV-V	12 (40.0)	3 (10.3)	8 (15.4)	4 (7.8)

^a^Table entries are No. (%)

### Morphologic model predicting unfavorable hemorrhage in pediatric bAVM

In the 49 pediatric patients with unfavorable hemorrhage, more than 85% had two or more risk factors: periventricular location, non-temporal location and long pial draining vein. The unfavorable hemorrhage survival was similar in children with or without one risk factor. In contrast, children harboring two or more predictors were at higher risk for earlier unfavorable hemorrhage (log-rank, *P* < .001). The estimated annual rate of unfavorable hemorrhage ranged from 0.7–1.0% per year for those with or without one risk factor, to 5.7% per year in cases harboring all three predictors (Fig. 2).

Notably, the majority of these patients (32/49; 65.3%) with unfavorable outcome after hemorrhage harbored bAVMs with periventricular and non-temporal location with or without long pial draining vein (Type I). Further analysis of this subgroup revealed that 18 of 32 bAVMs (56.3%) were supratentorial location involving corpus callosum, basal ganglia or thalamus adjacent to ventricle (Type Ia). 14 of 32 bAVMs (43.8%) were located in the frontal, parietal or occipital lobe or cerebellum adjacent to ventricle (Type Ib). Moreover, non-periventricular location (frontal, parietal, occipital lobe or deep location) or temporal lobe bAVMs with long pial draining vein (Type II) can also result in unfavorable hemorrhage (14/49; 28.6%) (Table 3).

18.5% (30/162) were Type Ia bAVMs, 60% of whom (18/30) had unfavorable hemorrhage during 367 patient-years follow-up, yielding an annual rate of unfavorable hemorrhage of 4.9%. 17.9% (29/162) were Type Ib bAVMs; During 280 patient-years follow-up, unfavorable outcome occurred in 48.3% of them (14/29) after hemorrhage, yielding an annual rate of unfavorable hemorrhage of 5.0%; 32.1% (52/162) were Type II bAVMs with an unfavorable hemorrhage rate of 26.9% (14/52) during 667 patient-years follow-up, yielding an annual rate of unfavorable hemorrhage of 2.1%. Generally, 93.9% of all unfavorable hemorrhage (46/49) occurred in bAVMs of these three types, and children with Type I lesions had unfavorable hemorrhage earlier than those with Type II lesions (log-rank, *P* < .001) (Fig. 2). The odds ratio for unfavorable hemorrhage of Type Ia and Type Ib bAVMs, compared with Type II bAVMs, was 2.37 (95% CI,1.10–5.14) and 2.45(95% CI, 1.08–5.53), respectively.

**Fig. 3:**
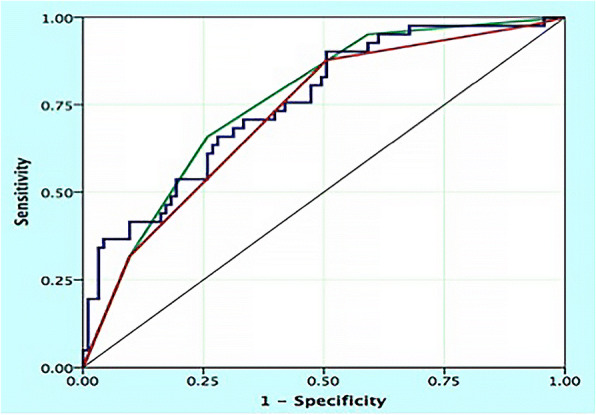
Receiver operating characteristic (ROC) analyses for unfavorable hemorrhage predictive models using all variants in regression model (blue curve), the bAVM classification system (green curve), and concise regression system including significant risk factors from the full model (red curve) (reference line shown in teal). The predictive accuracy of bAVM classification system (ROC areas of 0.77) was not less than that of full regression and concise regression model (ROC areas of 0.76 and 0.72, respectively; pairwise comparison of ROC curves, *P* = 0.87 for bAVM classification system versus full regression model and *P* = 0.08 for bAVM classification system versus concise regression model)

Proposed grading scales predicting post-hemorrhage outcome were evaluated using: (1) full variable regression model (10 variables); (2) concise regression model including significant risk factors from multivariable analysis (3 variables) and (3) bAVM classification (combining Type I subgroups). The AUROC, indicating the predictive accuracy of each model, was 0.76 (95% CI 0.67–0.84) for the full variable regression model, 0.72 (95% CI 0.63–0.81) for concise regression model, and 0.77 (95% CI 0.69–0.85) for bAVM type model (Fig. 3). There was no significant difference of predictive accuracy between bAVM classification model and full variable regression model or concise regression model (*P* = 0.87 and 0.08, respectively) The 5-fold cross validation showed similar estimates with AUROC of 0.70 (95% CI 0.54–0.85) for full logistic regression model, 0.74 (95% CI 0.68–0.80) for reduced logistic regression model, and 0.77 (95% CI 0.71–0.82) for bAVM classification model.

## Discussion

### Unfavorable hemorrhage in pediatric patients with bAVM

bAVMs are described as the underlying cause of the majority of childhood intracranial hemorrhage, and spontaneous ICH is generally considered to constitute the most devastating complication of bAVM, carrying a high burden of morbidity and mortality [1, 15, 16]. Although children with bAVMs are more likely to present with hemorrhage, a long-term study combining two bAVM cohorts found an annual subsequent hemorrhage rate of 2% in children, which was similar to the risk in adults [1]. Previous hospital- or population-based cohort studies revealed an overall case-fatality of 11–29% and severe disability rate of 14–38% after bAVM hemorrhage in adult [16–18]. Recent studies of children with untreated bAVM revealed that severe neurological deficit occurred in 22% of all patients and 30–42% of those with hemorrhage [5–7]. Therefore, bAVMs rupture is not always with unfavorable outcome or life-threatening. Furthermore, the presence of hemorrhage did not worsen the long-term neurological outcomes, while the children’s neurological function after hemorrhage could influence the long-term outcomes after treatment [5]. Although many studies aimed at identifying morphologic features associated with the risk of subsequent bAVM hemorrhage have been performed, only a few studies have specifically been conducted to assess the unfavorable hemorrhage risk in pediatric patients [5, 19–21].

In our cohort, we defined the hemorrhage as unfavorable when it is life-threatening (requiring emergent invasive intervention) or with disabling neurological deficit with mRS > 3 within 24 h after hemorrhage. We observed unfavorable hemorrhage in 30% of all children and 50% of those with ruptured bAVM, which are relatively congruent with previous reports [5, 6]. The annual rate of unfavorable hemorrhage in this pediatric cohort (2.5%) is higher than that quoted in other study of patients of all ages (1.4%) [22].

### Predictive model for unfavorable hemorrhage risk in pediatric bAVM

Although previous studies identified that a rupture history is the most significant risk factor for future hemorrhage [23], we hope to find predictive models based on features independent of rupture history, which could be used to predict the unfavorable outcome before AVM rupture. In this study with follow-up, we established a morphologic model predictive of unfavorable hemorrhage risk in children with bAVM. bAVM were categorized into three main types: Type I were based on periventricular and non-temporal location, with unfavorable hemorrhage of 50–60% and an annual rate of unfavorable hemorrhage of 5.0%; Type II were with long pial draining vein, which were based on non-periventricular location (frontal, parietal or occipital lobe or deep location) or temporal lobe, with unfavorable hemorrhage of 27.9% and an annual rate of unfavorable hemorrhage of 2.1%; Type III were based on non-periventricular location or temporal lobe without long pial draining vein, with very low rate of unfavorable hemorrhage.

The anatomic characteristics of brain might explain this morphologic model. Unfavorable neurological deficit usually involves impairment of fiber tract and deep nucleus. A non-temporal periventricular location is a deep located area consisting of aggregation structures of vital nucleus including thalamus, basal ganglia and hypothalamus, and important fiber tract such as corticospinal tract and arcuate fasciculus. Hemorrhage in non-temporal periventricular location could cause major disruption of the aforementioned nucleus and/or fiber tract. A recent study revealed that the plasticity of motor cortex on BOLD fMRI doesn’t prevent post-operative motor deficits, while the plasticity of motor fiber on preoperative DTI was correlated with neurological outcomes [24]. These findings suggest that the fiber tract might be more important in predicting neurological function than eloquent cortex, considering the distinct plasticity pattern and recovery capability after injury. Therefore, a non-temporal periventricular location might be a better alternative to define the “eloquence” of the brain than “deep location” and “eloquent region” which do not include important supratentorial fiber tracts.

Although an unfavorable neurological deficit might also occurred in a ruptured bAVM in temporal lobe, it is usually associated with language cortex and large hematoma contributing to temporal lobe hernia. However, our present study excluded those cases without radiologic or histologic diagnosis of bAVM, therefore, we might not include some ruptured bAVM with catastrophic temporal hernia but without a chance to confirm the diagnosis of bAVM through radiological methods or surgical specimen. For those temporal bAVM involving language cortex, previous studies suggested that language function reorganization is more commonly in the Wernick region [25] and we employed the mRS score to evaluate the neurological deficit in the present study, thus the language dysfunction might not be measured in details. Therefore, the present model only focused on predicting bAVM hemorrhage with disabling neurological deficit of extremities and life-threatening conditions, and the result should be interpreted with cautious in bAVM located in anterior-medial temporal lobe adjacent to cistern and Wernick region.

In the present study, we found a long draining vein was also a predictor for unfavorable hemorrhage. Our previous data suggested that a restricted venous outflow might be associated with higher hemorrhage risk of bAVM [26]. Recent quantitative studies of bAVM hemodynamics also found that ruptured bAVMs have significantly prolonged drainage times compared to unruptured bAVMs, indicating restricted bAVM venous drainage and increased intra-nidus pressure in ruptured bAVMs [27]. Actually, a long draining vein suggested both deeper bAVM location (distal to the superficial dura sinus) and more likelihood of venous restriction during the long draining course. Therefore, a long draining vein might represent a combination of a deep location and high venous outflow resistance, and be associated with higher risk of bAVM hemorrhage and injury of deep hematoma.

We suggested a subgroup in Type I bAVMs: Type Ia and Ib, considering different Spetzler-Martin grade and distinct treatment recommendation for these two subtypes. 70.8% of Type Ib lesions were classified as Spetzler-Martin Grade I-II (low-grade), for which microsurgical resection could be an effective treatment [28]. In contrast, only 24% of Type Ia lesion were Grade I-II (Table 3). With higher risk for unfavorable hemorrhage and more low-grade lesions, children with Type Ib bAVMs might benefit from treatment. Cautious follow-up might be recommended for children with untreated Type Ia lesions. Our data suggested that this bAVM classification system was a good predictor of post-hemorrhage outcome in children with bAVM. However, the validity of this stratification system should be further evaluated in future studies.

This study was limited by its sample size, single-institutional population. The annual unfavorable hemorrhage was based on an assumption and should be interpreted with caution [29]. The neurological deficit of unfavorable hemorrhage in this study was mainly measured through mRS scale, while further studies should analyze more conditions (for example, aphasia and cognitive function impairment) affecting children’s quality of life.

## Conclusions

Careful evaluation of nidus location and venous drainage is recommended to predict life-threatening and disabling hemorrhage in children with bAVM.

## Abbreviations

bAVM: Brain arteriovenous malformation
CI: Confidence interval
CSF: Cerebrospinal fluid
GCS: Glasgow Coma Scale
HR: Hazard ratio
ICH: Intracranial hemorrhage
mRS: Modified Rankin Scale
OR: Odds ratio
